# Inequalities in the uptake of weight management interventions in a pragmatic trial: an observational study in primary care

**DOI:** 10.3399/bjgp16X684337

**Published:** 2016-02-24

**Authors:** Amy L Ahern, Paul Aveyard, Emma J Boyland, Jason CG Halford, Susan A Jebb

**Affiliations:** MRC Human Nutrition Research, Cambridge.; Nuffield Department of Primary Care Health Sciences, University of Oxford, Oxford.; Department of Psychological Sciences, University of Liverpool, Liverpool.; Department of Psychological Sciences, University of Liverpool, Liverpool.; Nuffield Department of Primary Care Health Sciences, University of Oxford, Oxford.

**Keywords:** healthcare disparities, obesity, primary care, weight loss

## Abstract

**Background:**

Primary care referral to a commercial open-group behavioural weight-loss programme is a cost-effective intervention, but only 10% of patients receiving this intervention are male.

**Aim:**

To explore whether observed biases in participation in these interventions reflect biases in the uptake of the invitation to participate.

**Design and setting:**

Comparison of invited population and recruited participants in a multicentre randomised controlled trial of primary care referral to a commercial open-group behavioural weight-loss programme in England (WRAP [Weight loss Referrals for Adults in Primary care]).

**Method:**

Between October 2012 and February 2014, participants were recruited through 23 primary care practices in England; 17 practices provided data on the characteristics of invited participants.

**Results:**

Females were twice as likely as males to enrol in the trial (odds ratio [OR] 2.01, 95% confidence interval [CI] = 1.75 to 2.32). However, the proportion of males was threefold higher than seen in routine primary care referrals or similar trials that invited patients opportunistically. People from less deprived areas were more likely to enrol than those in more deprived areas (OR 1.77, 95% CI = 1.55 to 2.03). Older patients (≥40 years) were more likely to enrol than younger patients (OR 1.60, 95% CI = 1.34 to 1.91).

**Conclusion:**

Males, younger people, and those from more deprived areas were less likely to take up the invitation to participate in this trial. The gender bias was smaller than observed in routine practice, suggesting that a substantial proportion of the inequity observed previously is a consequence of bias with regard to the offer of intervention. This study suggests that a simple way to overcome much of the gender bias is to write to patients who are overweight and offer referral. Uptake of the invitation to participate was lower in groups of lower socioeconomic status suggesting the need to preferentially offer referrals to this group to reduce health inequalities and for research to explore barriers to uptake.

## INTRODUCTION

There is now good evidence that for people who are overweight, referral to a commercial open-group behavioural weight-loss programme is a cost-effective intervention for use in primary care.^1^ However, there remains a perception that these interventions only serve a subset of the population; namely middle-class, middle-aged women.^2^ Demographic biases in participation in weight-loss interventions offered through primary care may contribute to health inequalities, but there has been little systematic study of how these biases arise.

The prevalence of obesity is similar in males and females,^3^ but male participants are under-represented in randomised controlled trials of behavioural weight-management interventions^4^ and are less likely to have received treatment for obesity in clinical practice.^5^ In audits of commercial weight-loss programmes accessed through primary care, ∼90% of participants were female.^6^,^7^ Data on the effect of social status are less clear. There is a social gradient in the prevalence of obesity, particularly in women, such that people who are of a lower socioeconomic status (SES) are more likely to be obese than those of a higher SES.^3^ An audit of routine clinical data on obesity treatment in primary care suggested that patients from more deprived areas were more likely to receive treatment for obesity.^5^ However, population-based survey data suggest that those from more deprived areas are less likely to use commercial open-group behavioural weight-loss programmes than those from less deprived areas.^2^ There are no data on the SES distribution of people participating in these programmes when a referral is offered by the primary care provider at no cost to the individual. Qualitative data suggest that when GPs offer referral to a commercial programme, this can increase the perceived legitimacy of the intervention for those who may ordinarily perceive these types of programme as ‘not for them’, and that the provision of a ‘free’ weight management intervention can encourage participation in those who may not wish to spend family income on their own weight management.^8^

Given the diverse determinants of participation bias, it is important to distinguish between inequalities with regard to who is offered referral to a commercial weight-loss programme, which may reflect differences in practitioner behaviour or aspects of the referral system, and inequalities with regard to who accepts the offer, reflecting inter-individual differences among participants. For example, men may be less willing to participate in commercial open-group behavioural weight-loss programmes^9^ because they are less likely to visit the practice and thus less likely to be identified as likely to benefit from treatment,^10^ or because clinicians are also less likely to offer this type of intervention to men. In a randomised controlled trial of a commercial open-group behavioural weight-loss programme where GPs and nurses recruited patients opportunistically, the recruited participants were 12% male,^11^ a similar proportion to that seen in the audits of routine practice.^6^,^7^ However, in the Lighten Up trial, which recruited participants via a letter to all eligible patients and offered a variety of interventions, including these commercial programmes, the proportion of male participants was more than double this figure (30%).^12^ This comparison across studies suggests that there may be a bias with regard to who is offered this type of weight management support in routine practice. But there are currently no data on the uptake of intervention by gender in response to an offer from a GP.

**How this fits in**

Referral to a commercial open-group behavioural weight-loss programme is a cost-effective intervention for use in primary care, but only 10% of patients receiving this intervention through an NHS referral are male. The current study found that when all eligible patients in a practice were invited to participate in a trial offering this type of intervention, males, people from more deprived areas, and those aged <40 years were less likely to participate. However, the proportion of male participants was three times that seen in practice and in other trials that recruited opportunistically. This suggests that a substantial proportion of the gender inequality observed in NHS referrals and many clinical trials is a consequence of practitioner bias with regard to the offer of intervention. A simple strategy to reduce gender inequality with regard to participation in commercial open-group behavioural weight-loss programmes is to invite all eligible patients by letter.

The analysis reported here uses data from the WRAP (Weight loss Referrals for Adults in Primary care) trial, which recruited participants via an invitation sent to all eligible patients (adults with a body mass index [BMI] ≥28 kg/m^2^; identified by search of electronic records) in participating GP practices.^13^ By comparing the demographic characteristics of participants who enrolled in the trial to the characteristics of the invited population, any demographic biases with regard to which individuals took up the invitation from the GP to participate in this trial could be identified, and any bias with regard to who was offered the opportunity to participate could be controlled for.

## METHOD

### Design and setting

This study compared the invited and recruited populations of the WRAP trial. WRAP is a multicentre randomised controlled trial evaluating the clinical and cost-effectiveness of referral to a commercial open-group behavioural weight-loss programme (Weight Watchers^®^) for adults in England who are overweight or obese. Participants were recruited through 23 primary care practices in England who did not already have an existing contract with a commercial weight-loss provider. Recruitment took place between October 2012 and February 2014.

### Invited population

Each practice searched their electronic register and identified patients who were eligible to participate in the trial (age ≥18 years, BMI ≥28 kg/m^2^). GPs then excluded those who would be unsuitable for the study (for example, patients who were violent, terminally ill, or had a history of an eating disorder). Eligible patients were then sent a letter of invitation, signed by the GP, inviting them to participate in the trial. This letter explained that involvement in the trial was entirely separate to their usual GP care and could include referral to a commercial weight-loss provider at no cost to the patient. Patients were asked to contact a member of the research team if they were interested in participating.

### Recruited participants

Interested patients completed a telephone screening with a member of the research team. Exclusion criteria were: current or planned pregnancy in the next 2 years; previous or planned bariatric surgery; currently following a weight-loss programme (defined as a structured, prescribed, and monitored programme and not a self-regulated diet); unable to speak English or with special communication needs that would make it difficult to participate in the interventions offered without additional support.

Patients who were eligible at the telephone screening attended a baseline visit at the research centre, where their eligibility was confirmed, and they gave informed consent to participate in the study. The recruited population was defined as those eligible participants who were randomised to one of the trial arms.

### Outcome measures

On completion of the mail-out to patients, practices provided the research team with summary data on the gender, age, BMI, and ethnicity of the patients who had been invited.

At the baseline appointment, information on gender, age, and ethnicity was collected directly from participants using a questionnaire.

The postcode for each practice was used to calculate the Index of Multiple Deprivation (IMD) using the English indices of deprivation 2010 as markers of SES.^14^ The IMD ranks small geographical areas in the UK from the least deprived to the most deprived on seven indices: income, employment, health deprivation and disability, education, crime, barriers to housing and services, and living environment.

### Analysis

Demographic differences between invited patients and recruited participants were explored separately for gender, SES, and age using frequency tables. χ^2^ values and odds ratios (ORs) were calculated for each comparison. GP practices did not provide sufficient data on the ethnicity and BMI of those invited to enable a meaningful analysis of uptake by ethnicity or BMI.

## RESULTS

Across the 23 practices participating in the trial, 1269 participants were recruited. Sixty-eight per cent of participants were female, 90% were white British, with a mean age of 53.2 years (standard deviation [SD] 13.8) and a mean BMI of 34.5 kg/m^2^ (SD 5.2) (Table 1).

**Table 1.** Baseline characteristics of all recruited participants (*n* = 1269)

**Variable**	**Mean (SD)**
Age, years	53.2 (13.8)
Weight, kg	96.2 (17.4)
Height, cm	166.8 (9.1)
BMI, kg/m^2^	34.5 (5.2)

**Gender**	***n* (%)**
Female	862 (67.9)
Male	407 (31.9)

**Ethnicity**	***n* (%)**
Asian/Asian British	35 (2.8)
Black/black British	23 (1.8)
Mixed/multiple ethnic group	15 (1.2)
White/white British	1138 (89.7)
Other	15 (1.2)
Not stated/preferred not to say	43 (3.4)

*BMI* = *body mass index.*

Data on the gender and age of invited patients were provided by 17 practices (Table 2), which recruited 72% of the total study population. Practices not providing data reported problems with computer system changes or staff oversight in not recording the necessary information. There were no significant differences between practices that provided data and those that did not in terms of gender, age, and baseline weight of recruited participants. Data reported here is from the 17 practices that provided data. These practices invited 13 949 patients and recruited 910 participants (6.5%). The invited population was 52% female; 10 practices had an IMD score above the national median and seven practices had a score below the median.

**Table 2.** Characteristics of invited and recruited populations for the 17 practices that provided data

**Variable**	**Invited, *n***	**Recruited *n* (% of invited)**	**OR (95% CI)**
**Gender**			
Male	6785	300 (4.4)	–
Female	7164	610 (8.5)	2.01 (1.75 to 2.32)

**Practice IMD^a^**			
Above national median	7631	376 (4.9)	–
Below national median	6318	534 (8.5)	1.77 (1.55 to 2.03)

**Age, years**			
<40	3384	155 (4.6)	–
≥40	10 565	752 (7.1)	1.60 (1.34 to 1.91)

IMD = Index of Multiple Deprivation.

a *IMD calculated using practice postcode, higher IMD* = *more deprived location. OR* = *odds ratio.*

### Recruitment by gender

Practices invited 6785 males and 300 (4.4%) enrolled in the trial. They invited 7164 females and 610 (8.5%) enrolled. There was a significant association between gender and enrolment (χ^2^ [1 degree of freedom {df}] ] = 95.74; *P*-value<0.001), with female patients more likely than males to enrol in the trial in response to the invitation (OR 2.01, 95% CI = 1.75 to 2.32).

### Recruitment by socioeconomic status

The 10 practices with an IMD score above the national median (those in more deprived areas) invited 7631 patients and recruited 376 participants (4.9%). The seven practices with an IMD score below the national median (those in less deprived areas) invited 6318 patients and recruited 534 participants (8.5%). There was a significant association between practice IMD and enrolment (χ^2^ [1 df] = 61.62; *P*-value<0.001). Patients from practices in less deprived areas were more likely to enrol in the trial than those from more deprived areas (OR 1.77, 95% CI = 1.55 to 2.03).

### Recruitment by age

Practices invited 10 565 patients aged ≥40 years and 752 were recruited (7.1%); 3384 patients aged <40 years were invited and 155 were recruited (4.6%). There was a significant association between age and enrolment (χ^2^ [1 df] = 27.15; *P*-value<0.001). Patients aged ≥40 years were more likely to participate than those <40 years (OR 1.60, 95% CI = 1.34 to 1.91).

## DISCUSSION

### Summary

In the current study, males, people from more deprived areas, and those aged <40 years were less likely to enrol in this trial involving a commercial open-group behavioural weight-loss programme relative to females, those from less deprived areas, and older patients. Although male patients were less likely than females to enrol in this trial, the proportion of males enrolling (32%) was three times that seen in audits of referral to commercial open-group behavioural weight-loss programmes (∼10%).^6^,^7^ The proportion of men participating in the WRAP trial (32%) is similar to that seen in the Lighten Up trial (31%), which included referral to commercial behavioural weight-loss programmes in their interventions and also recruited by letter of invitation to all eligible patients suggesting this is a generalisable figure.^12^ It is more than double that observed in a trial of the same commercial programme where the practitioner invited patients opportunistically (13%).^11^ This suggests that a substantial proportion of the gender inequality observed in NHS referrals and many clinical trials can be attributed to inequalities in who is invited to participate in weight management interventions, and could be prevented by reducing this bias.

### Strengths and limitations

This unique dataset allowed the comparison of the demographic characteristics of people invited to participate in a commercial open-group behavioural weight-loss programme with those of people who accepted that invitation. This effectively separated bias with regard to who chooses to participate from bias with regard to who is offered the opportunity to participate, and gives an important insight into potential strategies to reduce inequalities. The finding that male participation can be increased through reducing inequalities in who is invited is drawn from cross-study comparisons; empirical confirmation using an experimental design may be warranted. This analysis is limited by the summary nature of the data available from practices about the invited population and not all practices provided data. In particular, insufficient data was provided to allow a meaningful analysis of uptake by ethnicity or BMI and further research should explore whether there are biases in uptake of referrals for these groups. It is also important to consider that uptake in the context of trial participation could differ from uptake in routine clinical practice and some people may be more willing to take part in the intervention outside of the trial. For example, people who work full time may find it difficult to take time off work for study visits and this could differentially affect men and younger adults. While research staff attempt to be flexible and offer out-of-hours appointments, this can be difficult to arrange. It is also not possible to say whether the biases observed here are specific to this type of intervention or common to all weight-loss interventions.

### Comparison with existing literature

Biases in participation in weight-loss interventions have previously been documented in clinical trials^4^ and routine practice.^5^ Concerns over low male participation in weight-loss interventions has led to the development of interventions that specifically target men, through links to sports clubs and a focus on masculinity,^15^ as well as the provision of men-only groups within existing programmes.^12^ There is growing evidence that some of these interventions can achieve weight loss, although there is not yet any evidence on whether they are more effective than existing interventions that are known to be effective in both males and females (or indeed whether these new interventions, often sports-related, targeted at men could also be effective for women).^16^ Men who do participate in commercial weight-loss interventions lose as much, if not more, weight than women. There is good reason to suppose that many men will find gender-specific interventions more appealing and will be more likely to participate in these than in more traditional commercial behavioural weight-loss programmes.^17^ However, not all men require this ‘masculine’ focus, and many men can and do lose weight through non-gender-specific interventions.^16^ An invitation from the GP may also legitimise attendance at a commercial open-group behavioural weight-loss programme for some males who had previously viewed these as a female domain.^8^

Providing alternatives to traditional weight loss programmes may be one way of increasing male participation, but these data suggest that a significant proportion of gender bias could emanate from who GPs think is suitable for a referral to these services rather than from men themselves and their willingness to participate. Other examples of practitioner bias have been seen in previous studies, including physicians being more likely to refer overweight women for weight-loss interventions than overweight men,^18^ and setting larger weight-loss goals for women who are obese than for men of the same size.^19^ Comparisons across studies suggest the gender bias in participation in commercial programmes can be considerably reduced by simply offering the intervention to everyone who is eligible.

The lower uptake rate in the more deprived areas suggests some bias in the willingness or ability of patients of lower SES to participate in commercial open-group behavioural weight-loss programmes. There is some evidence from survey data to suggest that people of lower SES are less likely to use these types of programmes than people of higher SES.^2^ However, data on the proportion of patients of lower SES participating in the NHS referral schemes to commercial providers, where the intervention cost is paid for by the NHS, are not currently available. Encouragingly, there is evidence from an audit of obesity treatment in primary care to suggest that primary care practitioners are more likely to offer some form of obesity treatment for patients of lower SES,^5^ perhaps in spite of the lower apparent likelihood of accepting the referral observed in this study.

Patients aged <40 years were less likely to take up the offer to participate in this trial. This age bias is not evident in the audit of the commercial referral scheme, where the proportion of patients <40 years is similar to that in the invited population.^6^ However, routine primary care data show that older people are more likely to receive some form of treatment for obesity.^5^ This could suggest that patients aged <40 years are less likely to take up the offer of obesity treatment, but are preferentially referred to commercial referral schemes, which mitigates this bias. Further research is needed to explore age-related biases in access and uptake of treatment for obesity.

### Implications for research and practice

British GPs are paid to maintain a register of patients who are obese, and the data from this study suggest that comparable numbers of males and females have a recorded weight that identifies them as obese, which mirrors national prevalence data. The current data also suggest that the gender bias in uptake of a commercial weight-loss programme is not sufficient to explain previously observed biases in participation of these programmes. Taken together, this suggests that GPs are equally likely to identify males and females as being overweight, but are less likely to offer male patients referral to a commercial weight management programme. This difference is important since referral to a commercial programme is the intervention with most evidence of effectiveness in primary care.^1^ This study suggests that a simple way to overcome a substantial proportion of the gender inequality observed in participation is to write to patients who are overweight and offer referral. There is a lower likelihood of referrals for obesity treatment to be accepted by lower SES groups, but some evidence that GPs may already be preferentially selecting lower SES groups for weight-loss interventions. This seems appropriate given the greater burden of avoidable morbidity and mortality faced by these groups. It is important to explore the impact that changes in referral practices may have on participation in obesity treatment programmes.
